# Forecasting Daily Volume and Acuity of Patients in the Emergency Department

**DOI:** 10.1155/2016/3863268

**Published:** 2016-09-20

**Authors:** Rafael Calegari, Flavio S. Fogliatto, Filipe R. Lucini, Jeruza Neyeloff, Ricardo S. Kuchenbecker, Beatriz D. Schaan

**Affiliations:** ^1^Department of Industrial and Transportation Engineering, Federal University of Rio Grande do Sul, Porto Alegre, RS, Brazil; ^2^Endocrine Division, Hospital de Clínicas de Porto Alegre, Federal University of Rio Grande do Sul, Porto Alegre, RS, Brazil; ^3^Emergency Department, Hospital de Clínicas de Porto Alegre, Federal University of Rio Grande do Sul, Porto Alegre, RS, Brazil

## Abstract

This study aimed at analyzing the performance of four forecasting models in predicting the demand for medical care in terms of daily visits in an emergency department (ED) that handles high complexity cases, testing the influence of climatic and calendrical factors on demand behavior. We tested different mathematical models to forecast ED daily visits at Hospital de Clínicas de Porto Alegre (HCPA), which is a tertiary care teaching hospital located in Southern Brazil. Model accuracy was evaluated using mean absolute percentage error (MAPE), considering forecasting horizons of 1, 7, 14, 21, and 30 days. The demand time series was stratified according to patient classification using the Manchester Triage System's (MTS) criteria. Models tested were the simple seasonal exponential smoothing (SS), seasonal multiplicative Holt-Winters (SMHW), seasonal autoregressive integrated moving average (SARIMA), and multivariate autoregressive integrated moving average (MSARIMA). Performance of models varied according to patient classification, such that SS was the best choice when all types of patients were jointly considered, and SARIMA was the most accurate for modeling demands of very urgent (VU) and urgent (U) patients. The MSARIMA models taking into account climatic factors did not improve the performance of the SARIMA models, independent of patient classification.

## 1. Introduction

Emergency department (ED) crowding results from mismatch between existing capacity and various input, throughput, and output factors. On such context, forecasting the demand may provide useful inputs for planning available resources [1]. ED demand prediction, expressed in terms of daily visits, has been assessed by different time-series approaches employed to develop forecasts models, with no supremacy of one method over others [2]. Studies compared several different methods for forecasting ED daily visits including ARIMA, SARIMA, multiple linear regression, time-series regression, and exponential smoothing [2–4].

Published literature assessing the impact of climate variables on ED demand (i.e., ED daily visits) provides conflicting results. While some studies indicate that climate variables such as mean daytime air temperature positively correlate with demand for ED services [4, 5], others show that climate variables add little predictive value to models of daily patient volumes [2, 6]. Temperature and calendar variables may provide different forecasting abilities, although in general forecasting accuracy of models including those variables tends to be better for the short-term horizon (7 days in advance) than for the longer term (30 days in advance) [2]. Several studies demonstrate that daily demand for ED is characterized by seasonal and weekly patterns [2, 4, 6, 7]. Daily patient's volumes show a weekly seasonal distribution, especially on Mondays [4, 6], although published studies demonstrate little variation in daily visits by month [2, 7].

In such context, accurate prediction of ED services' demands emerges as a powerful tool to support resources planning decisions [8, 9]. For example, long-term demand forecasts may be used to analyze infrastructure and personnel expansion plans [6], while short-term forecasts may support the operational planning of available resources on a daily basis [10]. The use of forecasting models to estimate the demand for ED services may improve the quality of the care offered to patients [9, 11]. ED managers may, for instance, identify the day in the week with the heaviest demand and align materials and personnel resources to attain better patient service levels [12]. The higher forecasting accuracy the better; therefore, factors that may influence demand in EDs should be considered as potential explanatory variables in the prediction models [13].

As reported above, studies have shown that demand forecasts in EDs may be improved by considering in the models factors such as day of the week and month and proximity to holidays [11, 12], in addition to climatic characteristics [2, 14, 15]; other studies, however, show the opposite [4, 16]. One explanation for that is the fact that each ED analyzed has its own characteristics. The effect of climate variables, for example, is dependent on the geographic location of the ED from which data were collected. In any case it is always advisable to test the influence of potential explanatory variables when developing an ED demand forecasting model [2]. Accurate forecasting according to categories of patient acuity (which indicates the degree in which healthcare services will be demanded to provided appropriate care to patients) may also provide a valuable tool for resources planning. In a study held in an ED in Singapore, attendances of lower acuity patients were significantly correlated with day of the week, month of the year, public holiday, and ambient air quality [14].

In this paper we analyze the performance of four forecasting models in predicting the demand for medical care according to patient classification using the Manchester Triage System (MTS) in the ED of a Brazilian University hospital.

## 2. Methods

### 2.1. Study Design and Setting

This is a retrospective study that uses historical data on medical care demands from an ED to develop and compare forecasting models able to predict future demands. The study was conducted at Hospital de Clínicas de Porto Alegre (HCPA), a 842 bed, tertiary care teaching public hospital in Southern Brazil. The HCPA's Ethical Committee has approved the study and authors have complied with the recommendations of the Declaration of Helsinki. Data were extracted from the hospital's management system. Patients' personal data were preserved.

### 2.2. Selection of Participants

The study was carried out in the HCPA's ED. The department operates 24 hours a day, 7 days a week, throughout the year. It receives patients from the city of Porto Alegre and its metropolitan region, corresponding to a population of approximately 2 million. Daily data on the number of patients who were admitted to the ED comprised the period starting on January 1, 2013, until May 31, 2015, including all patients processed between 00:00 and 24:00 hours. Medical care was provided to 57,128 individuals during that period.

Historical weather data were obtained from the Instituto Nacional de Meteorologia (INMET) [17], which monitors weather conditions 24 hours a day throughout the year in Brazil. We used weather information aggregated on a daily basis, to match with the dependent variable in the prediction models (i.e., ED daily demand). INMET methods comply with international standards defined by the World Meteorology Organization.

### 2.3. Study Protocol

Input data were the daily sum of patients that received care in the ED. Classification of patients that seek medical assistance at the ED follows the MTS criteria [18]. This system organizes care delivery into five levels of priority. Categories are identified by colors, case description, and the estimated time to service (ETS); they are (i) red: emergency (EM), needing immediate assistance in emergency service, ETS = 00 minutes; (ii) orange: very urgent (VU), need for care in emergency service, ETS ≤ 10 minutes; (iii) yellow: urgent (U), patient displaying clinical condition that enables waiting for care in emergency service, ETS ≤ 60 minutes; (iv) green: standard (ST), patient who may be directed to outpatient service such as ambulatory consultation, ETS ≤ 120 minutes; and (v) blue: nonurgent (NU), patient who should be directed to outpatient service such as ambulatory consultation, ETS ≤ 240 minutes. The color white is also used to indicate patients that were not classified and for which no case description or ETS is available.

The database of ED visits was divided into two periods, one for training, and the other for test. The first period, from January 1, 2013, to March 2, 2015, was used to analyze data and test forecasting models (training set). The second period, from March 3, 2015, to May 31, 2015, was used in the validation of models and for accuracy checking (postsample forecasting set). Approximately 94% of the patients triaged in the ED in the two periods were classified in categories VU or U. We analyzed series comprised of patients of all categories, in addition to individual series containing only patients in categories VU or U.

The postsample forecasting set was divided into three test intervals of 30 days: (i) 03/03/15 to 04/01/15, (ii) 04/02/15 to 05/01/15, and (iii) 05/02/15 to 05/31/15. Model accuracy was evaluated considering forecasting horizons of 1, 7, 14, 21, and 30 days. Forecasting models had their accuracy initially verified for the first interval of 30 days. Then, the first test interval was added to the training set, models were recalibrated considering the new data, and accuracy was verified for the second test interval. The same procedure was repeated when testing the third interval. The idea here was to continuously update the training set with more recent demand values.

### 2.4. Data Analysis

Considering that the superiority of a given forecasting model over others depends on the data under analysis [19, 20], we chose to evaluate the performance of several models and to search for explanatory variables that could influence the demand for care in the ED. We evaluated the performance of four forecasting models based on the analysis of time series of demand data. They comprised (a) simple seasonal exponential smoothing (SS), (b) seasonal multiplicative Holt-Winters (SMHW), (c) seasonal autoregressive integrated moving average (SARIMA), and (d) multivariate autoregressive integrated moving average (MSARIMA). The MSARIMA model was tested incorporating climatic and calendrical influencing factors as independent variables, as shown in Table 1. Models (a) to (c) use as input data only the time series of past demands, while model (d) tests the benefits of including climatic and calendrical factors on the prediction accuracy. All analyses were performed using PASW Statistics 18 and Minitab 14.

The first and second models tested are based on exponential smoothing coefficients. Such models are able to capture systematic variation in the time series due to seasonality and/or trend, as well as sudden changes in the demand pattern, which are common in ED data [4]. On that study, models that best fitted the data were the simple seasonal exponential smoothing (SS), and the seasonal multiplicative Holt-Winters (SMHW) [4]. SMHW presents two variations: the additive model form is preferred when seasonal variations are constant through the series, while the multiplicative model form is preferred when seasonal variations are changing proportional to the series' level [7, 21, 22]. In our work, multiplicative form displayed a better fit to the data, with smaller associated prediction errors.

SARIMA, an extension of the traditional ARIMA (autoregressive integrated moving average) model that accounts for seasonal components, was the third method analyzed. The model captures the behavior of demand variables using historical data from the time series, being the one most widely used in healthcare-related forecasts [2, 15, 23]. The SARIMA model is described by seven parameters: (*p*, *d*, *q*)  (*P*, *D*, *Q*)_*s*_, where *p* represents the order of the autoregressive factor (AR), *d* represents the order of differentiation required to reduce nonstationarity in the data (I), and *q* represents the order of the moving-average model (MA). Parameters *P*, *D*, and *Q* are analogous to *p*, *d*, and *q*, however describing the seasonal portion of the model; finally, *s* represents the seasonal* lag *[24]. Definition of the best values for the model parameters may be carried out analyzing the autocorrelation function (ACF) and the partial autocorrelation function (PACF) plots. The models that offered the best fit to the training time series of historical medical care demands in the ED were (1,0, 4)  (0,1, 1)_7_ when all patients were considered, (0,0, 4)  (1,0, 1)_7_ considering only VU category patients, and (1,0, 2)  (1,0, 1)_7_ considering only U category patients.

An extension of SARIMA, the MSARIMA model, was also tested to predict ED patient visits. MSARIMA incorporates independent (explanatory) variables to SARIMA in search of a better characterization of the demand time series [14, 15]. In our study ED demand was used as dependent variable and climatic and calendrical factors as independent variables. In addition to the climate variables presented in Table 1, we tested the influence of those same variables in a 1- to 7-day lag. Thus, a total of 64 climate variables and 2 calendrical variables were tested as independent variables in the MSARIMA model; only those displaying *p* values ≤ 0.05 were kept.

Accuracy of the prediction models was measured comparing actual and predicted values during the test period. For that, we calculated the mean absolute prediction error (MAPE) for each forecasting horizon (1, 7, 14, 21, and 30 days). Being a scale-independent measure, MAPE allows comparison of results from different models applied to different time series. The statistic represents the average of absolute differences between predicted and observed values, given as a percentage; a small MAPE value indicates a model well fitted to data. Consider a series of *m* predicted values (${\hat{y}}_{1},{\hat{y}}_{2},\ldots ,{\hat{y}}_{m}$) and the corresponding observed values (*y*_1_, *y*_2_,…, *y*_*m*_); the MAPE statistic is calculated as follows:

(1) $$\begin{array}{c} MAPE=\left(\;\frac{1}{n}\overset{n}{\underset{t=1}{\sum }}\left|\;\frac{\left(y_{t}-{\hat{y}}_{t}\right)}{y_{t}}\;\right|\;\right)\times 100. \end{array}$$

## 3. Results

During the interval considered in this study 57,128 patients sought medical care in the ED, being 51,046 during the 755 days that comprise the training period and 6,082 during the 90 days that comprise the test period. On average, 68 patients sought medical care daily, ranging from 26 to 119. During the same interval the average temperature was 21.08°C, varying from 0°C to 40.6°C, with an average daily range of 9.68°C.

**Figure 1:**
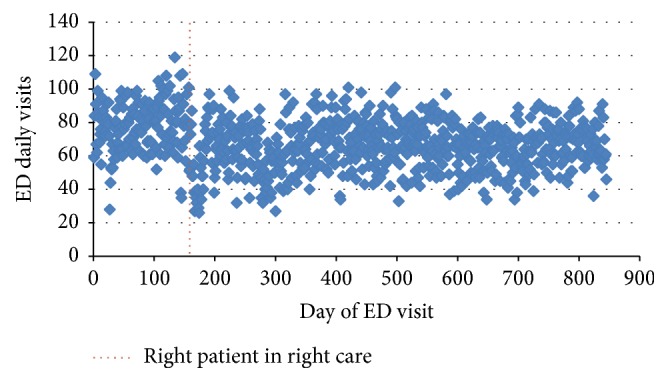
Scatter plot of total emergency department (ED) visits.

The scatter plot of the daily demand for medical care in the ED (Figure 1) displays a change in the series level around day 165 (July 14, 2013). That corresponds to the beginning of a new mechanism, entitled “right patient in the right place,” for referral of patients with minor acute illnesses (low acuity) to retail clinics and urgent care centers. The protocol was implemented as a partnership between the hospital and Porto Alegre's Health Department. After the new protocol was adopted the demand time series remained stationary, with no visible trends.

**Figure 2:**
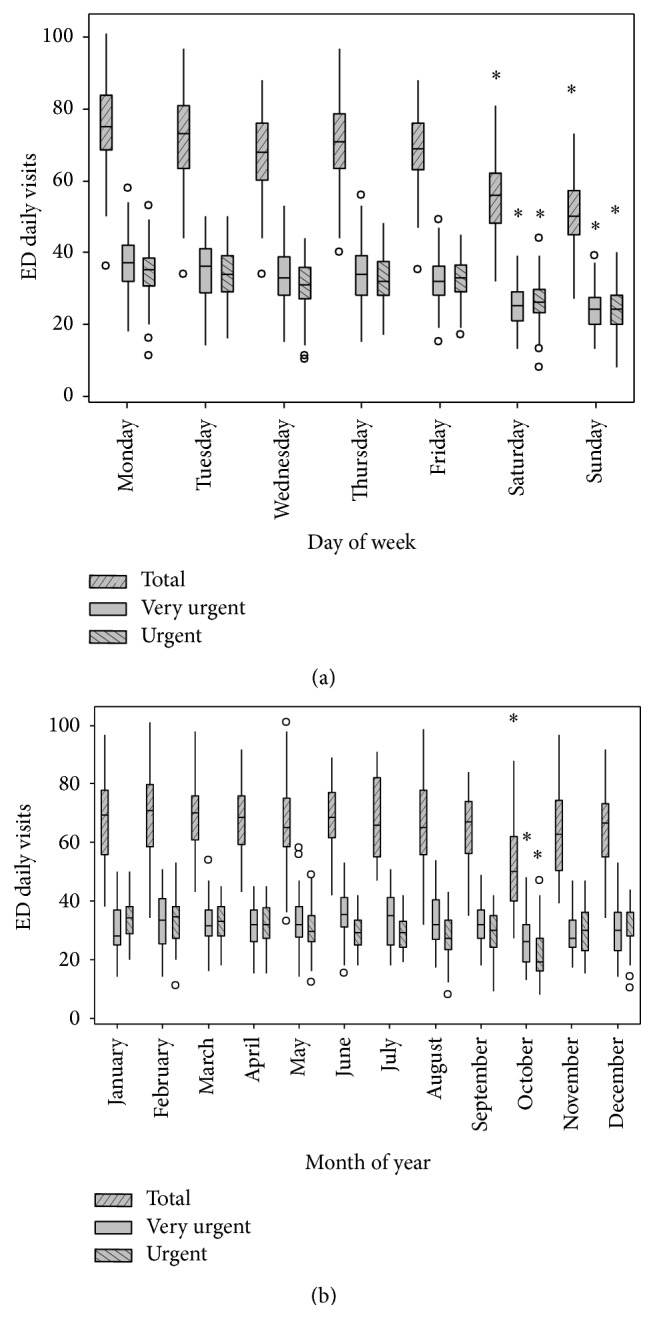
Demand variations within week (stratified per day) and year (stratified per month). ^*∗*^ *p* ≤ 0.05 (ANOVA, repeated measures).

Figure 2 presents demand variations within the week (Figure 2(a)) and the year (Figure 2(b)) in the form of box-plots. One-way ANOVAs were used to detect differences in weekdays and months, yielding the results in Figure 2. Demand is at its peak on Monday, followed by decrease until Wednesday; on Thursday it rises again, decreasing steadily until Sunday. However, statistically significant differences could only be found between weekdays and weekends.

To analyze variations in ED demands throughout the year (Figure 2(b)) we used the data series starting in August 2013, when the series mean stabilized after introducing the new mechanism for referral of patients with minor acute illnesses to retail clinics and urgent care centers. We also removed from the analysis the month of October 2014, when maintenance carried out in the department's air conditioning system led to an atypical reduction in demand during a period of 15 consecutive days. There were no differences in demand throughout the months, except for October, assigning to it the fact that only 2013 data were used to analyze this month. We also checked for differences in demand in holidays and regular weekdays; no differences were found.

Table 2 gives each model's MAPE value, considering all patients (TOTAL), and those in the very urgent (VU) and urgent (U) categories, and 5 different forecasting horizons (1, 7, 14, 21, and 30 days). In general, the shorter the horizon, the more accurate the forecast. Models' performances varied according to MTS patient classification, such that SS showed the best performance when all patients were considered, and SARIMA was the most accurate for modeling demands of high acuity (VU and U patients). Parameters of models presenting best fit to each patient category are given in Table 3. The MSARIMA models taking into account climatic factors did not improve the performance of the SARIMA models, independent of patient classification. In other words, the best MSARIMA models for every combination of patient category and horizon did not include climatic variables. When that is the case, MSARIMA reduces to SARIMA yielding identical MAPE values, and we chose SARIMA as best models.  Table 4 gives the *p* values for all climatic factors included in the models, for different time lags.

## 4. Discussion

In this study we analyzed the performance of four forecasting models in predicting the demand for medical care in an ED, testing the influence of climatic and calendrical factors on demand behavior. To the best of our knowledge, our study innovates by stratifying the demand time series according to patient acuity, according to the Manchester Triage System's (MTS) classification. In opposition to other studies that investigate the use of forecasting techniques to predict demand for medical care in EDs [4, 15, 23], we analyzed data from an ED that only provides care to high complexity cases.

A study held in an ED in Singapore assessed forecasting models where patients where stratified into three acuity categories (i.e., P1, P2, and P3), with P1 being the most acute and P3 being the least acute. P1 attendances did not show any weekly or yearly periodicity and were only predicted by ambient air quality. P2 and total attendances showed weekly variation and were predicted by holidays. P3 attendances correlated with day of the week, month, holiday, and ambient air quality. Higher total attendances on Monday were contributed mainly by P2 and P3 cases, while higher attendances on Sunday were essentially P3 cases [14]. Although resembling our study in that acuity of patients was taken into account when building forecasting models, the use of a local acuity scale by Sun et al. [14] does not allow us to directly compare their results with ours. Moreover, it is clear, considering the number of patients belonging to each category, that cases in the study of Sun et al. were less complex than those consulting our ED.

Regarding model accuracy, SS yielded the smallest MAPE values, independent of the forecasting horizon chosen, when all MTS classes of patients were considered in the demand series. For a 1-day horizon, SS yielded a MAPE of 2.91%, deemed excellent if compared to results obtained by Jones et al. [4]; for all remaining horizons investigated, the model yielded an average MAPE of 11%, which is aligned with results reported in similar works [2, 4]. Despite their complexity, the ARIMA models displayed the poorest performance in modeling ED demands, when all classes of patients were considered. As reported by Marcilio et al. [2], Tandbgerg and Qualls [3], and Jones et al. [25], depending on the characteristics of the demand time series analyzed simpler models, may lead to more accurate results.

As expected, models that forecasted the ED demand from patients classified as VU and U displayed larger MAPE values, in view of the smaller number of patients in the series and the larger variability. For those classes of patients, the model with the best performance was the SARIMA, except when VU patient demands where predicted for a 7-day horizon; in that case, SHWM yielded the smallest error (see Table 2). For a 1-day forecasting horizon it was possible to obtain a MAPE value of 7.19% for VU patients, and 3.98% for U patients, which are deemed acceptable and useful to support next day's managerial decisions in the ED. For larger forecasting horizons (7 to 30 days) MAPE values were larger, varying from 16.82% to 17.21% for VU patients and 12.79% to 15.21% for U patients. Considering that those patients are the ones that demand ED resources more intensely and frequently require inpatient beds, focusing the analysis on those classes of patients, as done here, is extremely relevant in practice. It is important to note that the results above were similar to those reported in other forecasting studies carried out using data from EDs that provide specialized and complex care [2, 14].

Regarding external information analyzed and incorporated to the models, only the day of the week showed a systematic effect on ED demand, independently of the patient classification considered. Day of the week has an effect on demand, while month of the year has not, as displayed in Figure 2. The effect of holidays and days following holidays on ED demand was also not significant. Demand peaks verified on Mondays, and demand valleys on weekend were aligned with results reported by several studies on ED demand available in the literature [2, 6, 14, 25]. There is a strong decrease in demand on weekends that could contribute to the optimization of ED resources and workforce management.

Some climatic factors displayed significant correlation with demand series from different classes of patients (see Table 4). However, when comparing the MAPE performance of MSARIMA models (that take such factors into account) and SARIMA models (that do not take them into account), the latter systematically outperformed the former. That may be due to the fact that the ED under analysis provides medical care to critical emergency situations in a teaching hospital, which seem to be less affected by climatic conditions.

Table 4 shows that VU and U classes displayed significant time lag terms for all lag instances. However, the same was not true for models considering the total number of visits. That is justified by the lack of identifiable pattern in the arrival of cases whose acuity classification leads to redirecting of patients to other types of care (i.e., all patient classes, except VU and U). Those are highly variable cases which, although representing a small percentage of the total number of visits, interfere in the prediction models' structure.

It is known that using forecasting techniques to predict demand in EDs may contribute directly to the reduction both in the number of patients that leave the unit without being served and in the number of complaints made by patients [6]. In the present study it was observed that models fitted to the demand series that considers all classes of patients displayed smaller prediction errors than those fitted to classes of patients. In addition, it was verified that the best forecasting model when demand from all classes of patients is considered (SS) is different from the model type that best fitted demands from individual classes of patients (SARIMA). It is important to remark that SS models are easier to implement in practice and that its superior performance in modeling ED demand has been reported in the literature [7].

The comparison between forecasting methods presented here indicates that, in the high complexity ED investigated, SARIMA models provide the most accurate prediction of demand for medical care for patients classified as VU and U. In other words, the inclusion of climatic factors as independent variables in the prediction model does not increase its accuracy. In short, results in this study do not support the common belief that daily demand for ED medical care is influenced by climatic factors [2, 14, 15].

Finally, results of the present study shall be viewed in the perspective of tertiary teaching hospitals, where usually overcrowded EDs are substantially affected by frequent bed shortages and seasonal variation of the number of patients referred by medical specialties. On such context, short-term horizon forecasting models may provide valuable information not only to optimize patient flow, but also to establish interventions in order to maintain the smooth and timely flow of patients throughout hospital facilities.

## 5. Limitations

We investigated data from a single hospital located in a specific region of Brazil. The test period comprised 25 days of summer and the first period of winter and therefore may not reflect the full variations in climate. Thus, our results are valid for highest level EDs located in the same region or in regions with similar climatic characteristics. For more generalizable results, the study should be extended to regions with different climatic characteristics. Also, the study did not assess the frequency of patients left without seeing. The same recommendation applies to the sample of patients.

## 6. Conclusions

Our study confirms that daily demand for ED services is characterized by seasonal and weekly patterns. Furthermore, it indicates that time-series models can be used to provide accurate forecasts of high complexity ED patient visits, especially short-range forecasts horizons. Forecasting accuracy depends on the model employed, length of the time horizon, and classification of the patient at MTS. In our analyses, SS yielded the smallest MAPE values, independent of the forecasting horizon chosen, when all classes of patients were considered in the demand series. For VU and U classes of patients, the model with the best performance was the SARIMA, except when VU patient demands where predicted for a 7-day horizon; in that case, SHWM yielded the smallest error. Some climatic factors displayed significant correlation with demand series from different classes of patients but do not increase the accuracy of prediction when incorporated into the model.

## Figures and Tables

**Table 1:** MSARIMA explanatory variables.

	Variables	Explanation
Calendrical	Month	January, February, March, April, May, June, July, August, September, October, November, December
	Day of the week	Sunday, Monday, Tuesday, Wednesday, Thursday, Friday, Saturday

Climatic	Average temperature	Average temperature in Celsius
	Minimal temperature	Minimal temperature in Celsius
	Maximal temperature	Maximal temperature in Celsius
	Temperature gap	Maximum-minimum temperature in Celsius
	Rain	Amount of rainfall
	Air-velocity	Wind speed in meters/second
	Relative humidity	% relative humidity
	Insolation	Hours of insolation

**Table 2:** MAPE values for each method and 5 different forecasting horizons.

	Forecasting horizon	1	7	14	21	30
Total	MSARIMA	6.23%	12.01%	11.79%	12.29%	11.51%
	SARIMA	6.23%	12.01%	11.79%	12.29%	11.51%
	SS	2.91%^*∗*^	10.67%^*∗*^	10.67%^*∗*^	11.35%^*∗*^	11.07%^*∗*^
	SMHW	3.02%	10.80%	10.85%	11.54%	11.11%

VU	MSARIMA	7.19%	17.65%	16.89%	17.23%	17.21%
	SARIMA	7.19%^*∗*^	17.65%	16.89%^*∗*^	17.23%^*∗*^	17.21%^*∗*^
	SS	11.16%	17.79%	17.36%	18.13%	19.14%
	SMHW	9.40%	16.82%^*∗*^	16.89%	17.56%	18.18%

U	MSARIMA	3.98%	12.79%	15.71%	14.74%	14.54%
	SARIMA	3.98%^*∗*^	12.79%^*∗*^	15.71%^*∗*^	14.74%^*∗*^	14.54%^*∗*^
	SS	7.18%	15.72%	18.04%	16.41%	16.60%
	SMHW	5.57%	14.24%	16.56%	15.50%	15.57%

^*∗*^Models with best fit

MSARIMA: multivariate seasonal autoregressive integrated moving average; SARIMA: seasonal autoregressive integrated moving average; SS: seasonal exponential smoothing; SMHW: seasonal multiplicative Holt-Winters; VU: very urgent; U: urgent.

**Table 3:** Parameters of models with best fit in each patient category.

	Model	Coefficients	Lag	Estimate	SE	*t*	Sig.
Total	SS	Alpha (level)	NA	0.200	0.021	9.436	0.000
		Delta (Season)	NA	0.212	0.018	6.090	0.000

VU	SARIMA	Constant	NA	30.356	1.879	16.151	0.000
		MA	1	−0.170	0.035	−4.872	0.000
		MA	3	−0.166	0.035	−4.753	0.000
		MA	4	−0.94	0.035	−2.660	0.008
		AR (Seasonal)	1	0.983	0.008	119.03	0.000
		MA (Seasonal)	1	0.885	0.023	38.324	0.000

U	SARIMA	Constant	NA	32.274	3.702	8.717	0.000
		AR	1	0.913	0.032	28.349	0.000
		MA	1	0.576	0.049	11.769	0.000
		MA	2	0.140	0.041	3.412	0.001
		AR (Seasonal)	1	0.986	0.007	137.952	0.000
		MA (Seasonal)	1	0.905	0.022	41.077	0.000


SS: seasonal exponential smoothing; SARIMA: seasonal autoregressive integrated moving average; VU: very urgent; U: urgent; NA: not applied; SE: standard error; *t*: *t*-value; Sig.: significance; MA: moving average; AR: autoregressive.

**Table 4:** Significance of climate variables by classification of patients and for different time lags.

	Total	VU	U
Average temperature (0)	0.293	0.379	0.183
Minimal temperature (0)	0.007^*∗*^	0.64	0.000^*∗*^
Maximal temperature (0)	0.048^*∗*^	0.441	0.000^*∗*^
Temperature gap (0)	0.098	0.1	0.260
Rain (0)	0.178	0.534	0.001^*∗*^
Air-velocity (0)	0.060	0.462	0.000^*∗*^
Relative humidity (0)	0.593	0.309	0.020^*∗*^
Insolation (0)	0.477	0.243	0.458

Average temperature (1)	0.828	0.888	0.876
Minimal temperature (1)	0.016^*∗*^	0.903	0.000^*∗*^
Maximal temperature (1)	0.010^*∗*^	0.611	0.000^*∗*^
Temperature gap (1)	0.618	0.423	0.902
Rain (1)	0.942	0.627	0.248
Air-velocity (1)	0.031^*∗*^	0.49	0.000^*∗*^
Relative humidity (1)	0.359	0.144	0.719
Insolation (1)	0.211	0.079	0.388

Average temperature (2)	0.818	0.809	0.948
Minimal temperature (2)	0.096	0.453	0.000^*∗*^
Maximal temperature (2)	0.061	0.149	0.000^*∗*^
Temperature gap (2)	0.819	0.533	0.555
Rain (2)	0.829	0.547	0.266
Air-velocity (2)	0.169	0.081	0.000^*∗*^
Relative humidity (2)	0.272	0.033^*∗*^	0.755
Insolation (2)	0.773	0.346	0.426

Average temperature (3)	0.480	0.796	0.077
Minimal temperature (3)	0.392	0.078	0.000^*∗*^
Maximal temperature (3)	0.349	0.042^*∗*^	0.000^*∗*^
Temperature gap (3)	0.885	0.862	0.732
Rain (3)	0.699	0.496	0.100
Air-velocity (3)	0.591	0.01^*∗*^	0.000^*∗*^
Relative humidity (3)	0.639	0.236	0.410
Insolation (3)	0.356	0.335	0.445

Average temperature (4)	0.652	0.613	0.080
Minimal temperature (4)	0.522	0.024^*∗*^	0.000^*∗*^
Maximal temperature (4)	0.518	0.018^*∗*^	0.000^*∗*^
Temperature gap (4)	0.861	0.602	0.464
Rain (4)	0.761	0.063	0.005^*∗*^
Air-velocity (4)	0.771	0.004^*∗*^	0.000^*∗*^
Relative humidity (4)	0.461	0.016^*∗*^	0.072
Insolation (4)	0.884	0.863	0.753

Average temperature (5)	0.657	0.779	0.436
Minimal temperature (5)	0.652	0.058	0.000^*∗*^
Maximal temperature (5)	0.262	0.03^*∗*^	0.000^*∗*^
Temperature gap (5)	0.487	0.847	0.204
Rain (5)	0.767	0.268	0.039^*∗*^
Air-velocity (5)	0.678	0.011^*∗*^	0.000^*∗*^
Relative humidity (5)	0.876	0.237	0.094
Insolation (5)	0.677	0.884	0.634

Average temperature (6)	0.788	0.791	0.211
Minimal temperature (6)	0.485	0.034^*∗*^	0.000^*∗*^
Maximal temperature (6)	0.374	0.036^*∗*^	0.000^*∗*^
Temperature gap (6)	0.964	0.544	0.674
Rain (6)	0.468	0.36	0.019^*∗*^
Air-velocity (6)	0.786	0.005^*∗*^	0.000^*∗*^
Relative humidity (6)	0.570	0.164	0.009^*∗*^
Insolation (6)	0.891	0.779	0.798

Average temperature (7)	0.059	0.067	0.235
Minimal temperature (7)	0.191	0.116	0.000^*∗*^
Maximal temperature (7)	0.744	0.011^*∗*^	0.000^*∗*^
Temperature gap (7)	0.119	0.441	0.133
Rain (7)	0.152	0.888	0.004^*∗*^
Air-velocity (7)	0.679	0.006^*∗*^	0.000^*∗*^
Relative humidity (7)	0.913	0.134	0.022^*∗*^
Insolation (7)	0.774	0.857	0.549

(*x*) days of lags

^*∗*^ *p* < 0.05.
